# Biomechanical evaluation of different strain judging criteria on the prediction precision of cortical bone fracture simulation under compression

**DOI:** 10.3389/fbioe.2023.1168783

**Published:** 2023-04-13

**Authors:** Ruoxun Fan, Jie Liu, Zhengbin Jia

**Affiliations:** ^1^ Department of Traffic Engineering, Yangzhou Polytechnic Institute, Yangzhou, China; ^2^ Department of Aerospace Engineering, Jilin Institute of Chemical Technology, Jilin, China; ^3^ Department of Mechanical and Aerospace Engineering, Jilin University, Changchun, China

**Keywords:** cortical bone, compression, fracture, principal strain, equivalent strain

## Abstract

**Introduction:** The principal strain or equivalent strain is mainly used in current numerical studies to determine the mechanical state of the element in the cortical bone finite element model and then perform fracture simulation. However, it is unclear which strain is more suitable for judging the element mechanical state under different loading conditions due to the lack of a general strain judging criterion for simulating the cortical bone fracture.

**Methods:** This study aims to explore a suitable strain judging criterion to perform compressive fracture simulation on the rat femoral cortical bone based on continuum damage mechanics. The mechanical state of the element in the cortical bone finite element model was primarily assessed using the principal strain and equivalent strain separately to carry out fracture simulation. The prediction accuracy was then evaluated by comparing the simulated findings with different strain judging criteria to the corresponding experimental data.

**Results:** The results showed that the fracture parameters predicted using the principal strain were closer to the experimental values than those predicted using the equivalent strain.

**Discussion:** Therefore, the fracture simulation under compression was more accurate when the principal strain was applied to control the damage and failure state in the element. This finding has the potential to improve prediction accuracy in the cortical bone fracture simulation.

## Introduction

With a load-bearing role, the cortical bone structure bears the majority of the external load and protects the soft tissue from damage (Kumar and Ghosh, 2022). Both the instantaneous impact and continuous static loads may lead to structural damage and crack occurrence in the cortical bone structure, and even direct fracture in extreme cases (Cui et al., 2022; Rosa et al., 2022). Therefore, investigating the relationships among the cortical bone mechanical properties, damage and failure mechanisms, and different loading conditions is necessary to explore preventive measures to reduce the possibility of cortical bone damage (Nourisa et al., 2019). However, the achievement of these first requires accurate observation of the complete failure process in the cortical bone structure.

The failure process in the cortical bone structure includes a series of mechanical behaviors from elastic deformation to crack initiation, propagation, and final fracture (Salem et al., 2022; Snow et al., 2022). Due to the limitation of image development, observing these mechanical behaviors at the micro-level by experiment is difficult (Ural, 2021). Therefore, most studies simulate the failure process of the cortical bone structure using finite element (FE) analysis. Early numerical research predicted the fracture load in the cortical bone structure by developing a linear elastic fracture model (Stitzel et al., 2004; Ural and Vashishth, 2006). With the development of fracture mechanics, the elastic-plastic fracture model was used to perform cortical bone fracture, which simulated the failure process by describing the change in the mechanical behavior during crack propagation (Prasannavenkadesan and Pandithevan, 2021; Remache et al., 2020). The cortical bone fracture can also be simulated using the continuum damage mechanics theory and the extended finite element method. Both methods can simulate the complete failure process in the cortical bone structure by defining the crack initiation, softening model, and element failure, and can simulate both brittle and ductile fractures by setting different softening models (Ng et al., 2017; Soni et al., 2021; Yadav et al., 2021; Kumar et al., 2022).

The above numerical fracture models differed but shared one feature: the strain was regarded as an indicator to control the damage and failure state in the element. Many studies have considered that strain is more accurate in determining the mechanical state of bone elements than stress (Cohen et al., 2017; Lovrenic-Jugovic et al., 2020). However, different strain judging criteria were used among the fracture models discussed above. One part applied the principal strain as the element damage and failure criterion in the cortical bone FE model, while the other part used the equivalent strain (Ng et al., 2017; Remache et al., 2020; Soni et al., 2021; Kumar et al., 2022). Because no standardized strain judging criterion for cortical bone fracture simulation was provided, it is unclear which strain is more suitable for judging the element mechanical state in the FE models under different loading conditions.

Cortical bone is a transversely isotropic material with mechanical properties that vary depending on the orientation of the osteon (Li et al., 2013; Szabo and Rimnac, 2022). Meanwhile, the main difference between the equivalent strain and principal strain is whether the shear strain is considered, so the numerical variation process in the two types of strain is different under various loading conditions (Xia and Wang, 2001). Therefore, the applicable range of the two strains in judging the element mechanical state should differ in cortical bone fracture simulation under different loading conditions.

This study aims to explore a suitable strain judging criterion for simulating cortical bone fracture under compression. The compressive experiment on the rat femoral cortical bone specimen was first performed. Then, the cortical bone FE model was established based on the femur micro images, and the equivalent and principal strains were separately set as the judging criteria to simulate the failure process in the cortical bone structure. Finally, the predicted fracture parameters were compared with the corresponding experimental data, and the effects of applying different strain judging criteria on the prediction precision of cortical bone fracture were discussed, the outcome of which has the potential to improve prediction accuracy in cortical bone fracture simulation under compression.

## Materials and methods

### Compressive experiment

**FIGURE 1:**
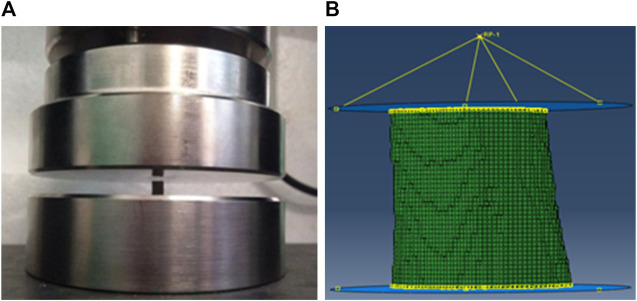
The schematic diagrams of the experiment and simulation on the rat femoral cortical bone structure **(A)** Compressive experiment; **(B)** Fracture simulation.

Six 3-month-old male Wistar rats were selected to obtain six right femurs. The location of the femoral mid-diaphysis was set as a benchmark, and the cortical bone specimen with a height of 5 mm was cut along the femoral shaft axis. All the cortical bone specimens were subjected to axial compression on the electronic testing machine, as shown in Figure 1A. The pressure head was compressed at a uniform speed of 1 mm/min to perform quasi-static loading, and the test started with an increase to 30 N, followed by a decrease to 0 N to ensure no slide on the specimen (Fang et al., 2019).

### Establishment of the cortical bone finite element model

The cortical bone micro images were obtained based on micro-CT scanning of the rat femurs. The images were first imported into the MIMICS software to reconstruct the geometry model of the femoral cortical bone structure, then the geometry model was imported into the ABAQUS software to establish the cortical bone FE model using the C3D8 element. To simulate the boundary condition in the compressive experiment, rigid circular plates were created above and below the cortical bone structure. The frictionless interaction was established between the lower rigid plate and the cortical bone structure, and the tangential behavior friction with a penalty coefficient of 0.2 was created between the upper rigid plate and the cortical bone structure (Hambli and Allaoui, 2013; Harrison et al., 2013). A reference point coupled to the upper rigid plate was set up above the model. An axial compression displacement of 0.5 mm was applied to the reference point, and all the degrees of freedom except the loading direction on the reference point were constrained. Meanwhile, all the degrees of freedom in the lower rigid plate were constrained. The boundary condition in the FE models can be seen in Figure 1B. Assigning the longitudinal and transverse elastic moduli for the FE model is necessary because the cortical bone is a transversely homogeneous material. After a previous study on this batch of rat femurs, i.e., the nanoindentation test on these cortical bone specimens, the average longitudinal and transverse elastic moduli of the cortical bone tissue were measured as 13,498 and 11,025 MPa, respectively, and the Poisson’s ratio was set to 0.3 (Zhang et al., 2015).

### Fracture simulation for the cortical bone finite element model

The fracture method based on continuum damage mechanics theory was adopted in this study. The implemented ABAQUS user’s subroutine (UMAT) was related to the damage variable *D* and used to perform fracture simulation. The stress–strain relationship after the onset of the damage can be expressed as (Cornin et al., 2022):

$$\sigma =C_{d}\varepsilon$$ (1)

$$C_{d}=\left(1-D\right)C$$ (2)

where σ is the stress tensor in the element of the FE model, $C_{d}$ is the damage elasticity matrix tensor in the element of the FE model, ε is the strain tensor in the element of the FE model, *D* is the damage variable in the element of the FE model, C is the elasticity tensor of the undamaged material in the element of the FE model.

The damage variable *D* was set to depend on the principal strain or equivalent strain. The change in the damage variable can be expressed as (Gaziano et al., 2022):

$$D=0\;\left(\varepsilon \leq \varepsilon_{f}\right);\;D=1-e^{\left(1-\frac{\varepsilon }{\varepsilon_{f}}\right)}\;\left(\varepsilon >\varepsilon_{f}\right)$$ (3)

Where ε is the principal strain or equivalent strain in the element of the FE model, ε_f_ is the critical failure strain in the cortical bone material.

**FIGURE 2:**
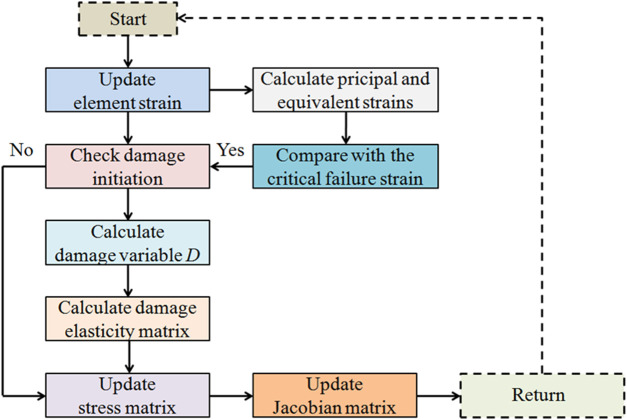
Detailed flow chart for simulating the progressive failure in the cortical bone structure using UMAT.

The critical failure strain in the femoral cortical bone of 3-month-old Wistar rats was obtained in our previous study, with a compressive failure strain threshold of 4.3% (Fan et al., 2016). As the compression condition was considered in this study, the compressive strain in the element was dominant, so the minimum principal strain was compared with the equivalent strain to determine the suitable strain judging criterion. In the process of compression simulation, the minimum principal strain and equivalent strain were separately compared with the critical compressive failure strain threshold of the cortical bone material to determine the element damage initiation. Element damage occurred when the minimum principal or equivalent strain exceeded 4.3%. In the damaged element, the damage variable *D* increased as expression 3), and the elastic modulus decreased as expression 2). The damage elasticity matrix was adopted to update the stress and the Jacobian matrix in the element. The Jacobian matrix, after the onset of the damage, could be described as expression 4) (Gao et al., 2018). The maximum value of the damage variable was set to 0.9 (Hambli et al., 2012). The element failed and lost its load-bearing capacity when the elastic modulus reduced to 10% of the original. The apparent failure occurred in the cortical bone structure when the failed elements accumulated to a certain degree. The flow chart of the fracture simulation can be seen in Figure 2. By comparing the numerical differences between the simulation results obtained by applying the minimum principal and equivalent strains, the strain judging criterion that is more suitable for simulating the fracture of the rat femoral cortical bone under compression can be determined.

$$\frac{\partial \sigma }{\partial \varepsilon }=C_{d}+\frac{\partial C_{d}}{\partial \varepsilon }:\varepsilon =C_{d}+\frac{\partial C_{d}}{\partial D}\times \frac{\partial D}{\partial \varepsilon }$$ (4)

## Results

### Mesh sensitivity analysis

**FIGURE 3:**
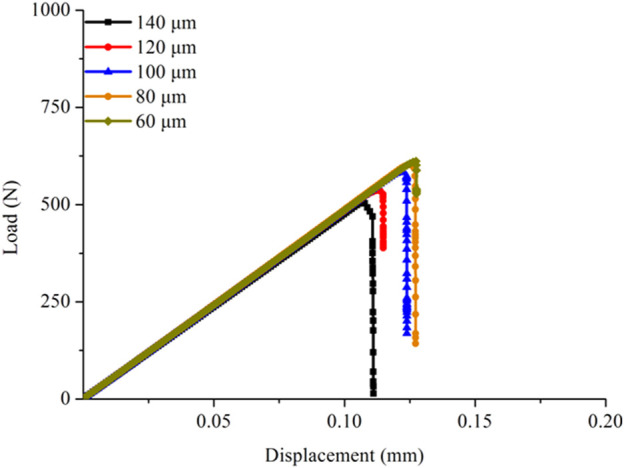
Mesh sensitivity analysis on the cortical bone FE models.

Mesh sensitivity analysis was first conducted to determine the suitable element size for the FE models. Five sizes (60, 80, 100, 120, and 140 μm) were used for creating five cortical bone FE models, and the fracture simulation was performed by applying the equivalent strain to observe the effects of different sizes on the failure process. Figure 3 shows that the fracture load increased with the fine mesh, indicating that the damage variable *D* rose faster for the large element size and led to a faster decrease in the structural stiffness. Meanwhile, when the element size was in the range of 60–100 μm, the shapes of the load–displacement curves did not differ much. The fine mesh was needed because the fracture method adopted in this study cannot make a crack in the element. Therefore, the element size was selected to 80 μm for creating the cortical bone FE models. The element number of the six established cortical bone FE models was in the range of 15,230–16,843 in this study.

### Comparison of the fracture patterns in the simulation and experiment

**FIGURE 4:**
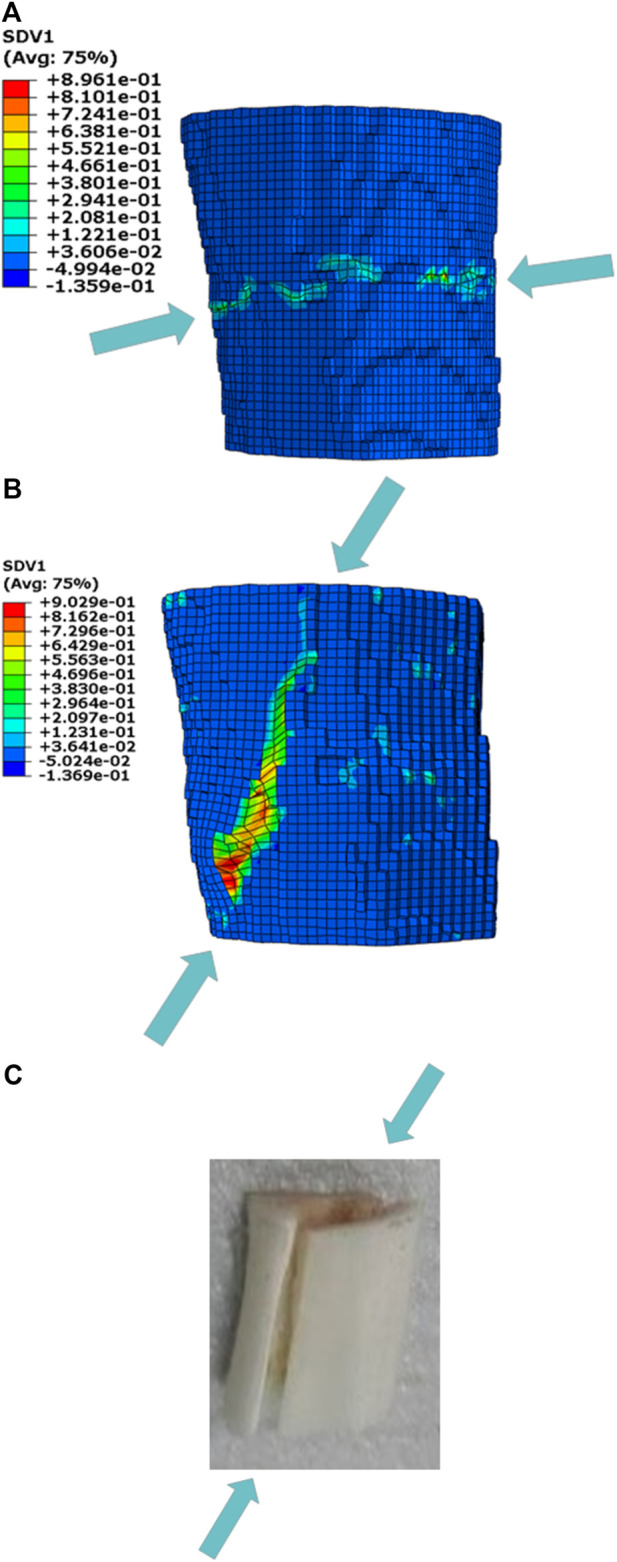
Comparison of the fracture patterns in the simulations and compressive experiment **(A)** Fracture simulation predicted by applying the equivalent strain; **(B)** Fracture simulation predicted by applying the minimum principal strain; **(C)** Compressive experiment.

Figure 4 compares the fracture patterns in the simulations and experiment. The simulation using the equivalent strain expressed a transverse slide-open fracture at the end of compression. The crack was produced in the middle of the structure and propagated in the direction of perpendicular to the compression load. In contrast, the simulation predicted by applying the minimum principal strain exhibited a longitudinal slide-open fracture, and the crack propagated along a certain angle to the loading direction. The comparisons of the fracture patterns stated that the simulation predicted by applying the minimum principal strain was in better agreement with the experimental results.

### Comparison of the predicted curves between the simulations and experiments

**FIGURE 5:**
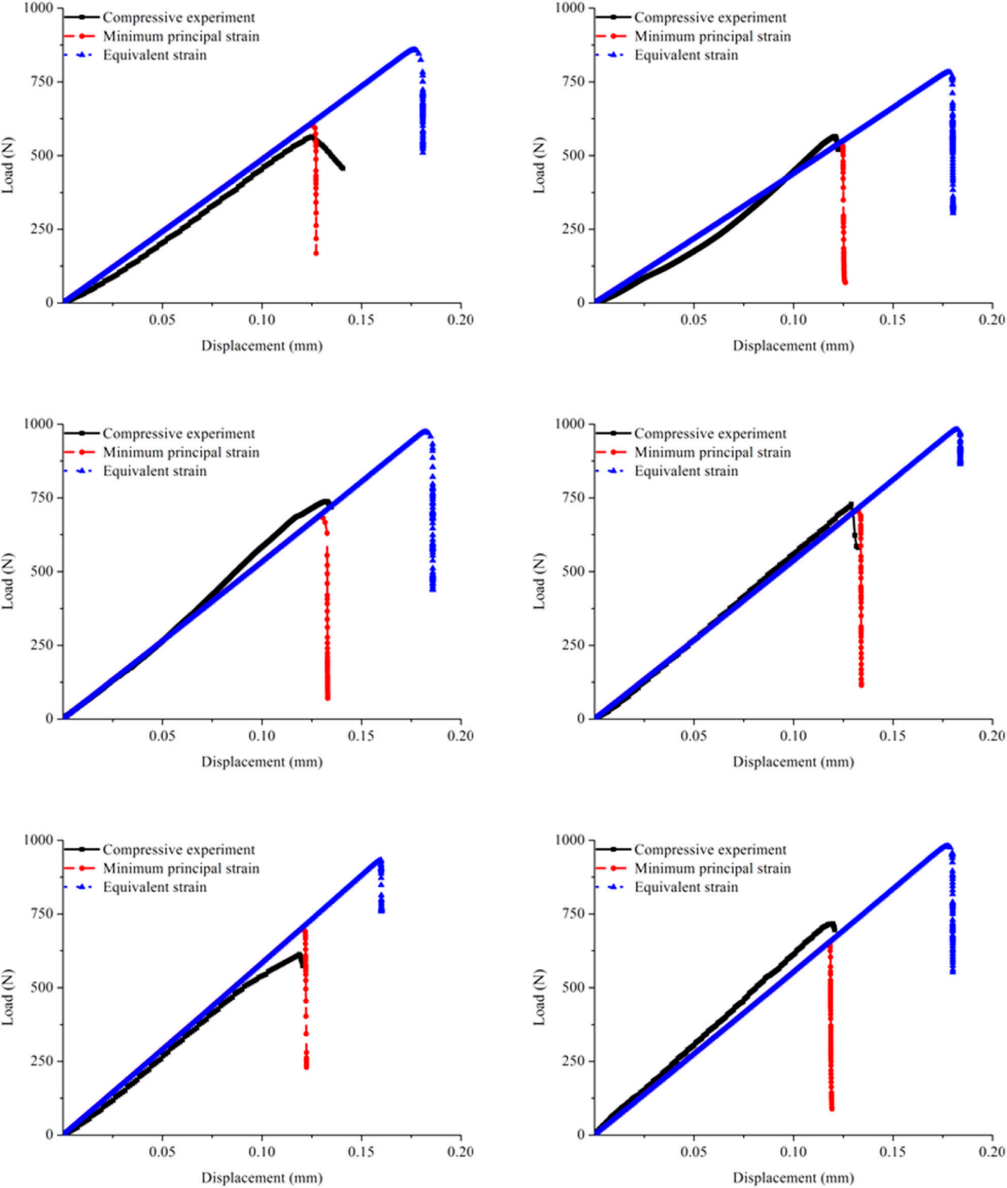
Comparison of the load–displacement curves in the six specimens and corresponding FE models obtained from the experiments and simulations separately using the minimum principal strain and equivalent strain.

Figure 5 shows the load–displacement curves obtained from the experiments and the corresponding simulations separately by applying the minimum principal and equivalent strains. The experimental curves exhibited that the quasi-brittle fracture occurred in the rat femoral cortical bone structure under compression. The yielding behavior was not appear after the elastic phase, and the apparent stiffness also hardly changed before the complete fracture. The simulation curves also exhibited quasi-brittle fracture in the cortical bone FE models. On the premise that the elastic moduli in the cortical bone FE models were all acquired from the nanoindentation test, the apparent stiffness predicted by the simulations using different strain judging criteria agreed with the experimental results.

The shapes of the predicted curves obtained by applying the two strain judging criteria were essentially similar, but the fracture parameters differed. At the beginning of loading, the curves coincided because the assigned elastic moduli were the same. With increasing compression, the simulation performed by applying the minimum principal strain first entered the fracture stage, while the simulation performed by applying the equivalent strain underwent a relatively long elastic phase before the complete fracture. Therefore, the fracture time in the cortical bone FE model predicted by applying the equivalent strain was remarkably later than that predicted by applying the minimum principal strain, which directly led to great differences in the fracture load. Furthermore, the comparison with the experimental curves showed that the simulated results obtained by applying the minimum principal strain were close to the experimental results, while the results predicted by applying the equivalent strain were different from the experimental data.

## Discussion

Adopting different strain criteria in judging the element mechanical state in the FE model may lead to differences in the predicted results, even for the same structure (Russell et al., 2012; Yavari et al., 2013). To explore the suitable strain judging criterion in compression conditions, this study simulated the cortical bone fracture under compression load based on the continuous damage mechanics theory. The prediction accuracy of the fracture simulation using the minimum principal and equivalent strains was first determined, then the reasons for the differences in the mechanical responses of the same cortical bone structure under the two simulations were exhibited and explained.

It was observed experimentally that quasi-brittle fracture occurred in the femoral cortical bone structure under compression in this study. Based on the literature, for brittle fracture, the consistency of the failure process can be verified by comparing the strength limit, the apparent failure strain, and the apparent stiffness in the elastic phase (Dall'Ara et al., 2013; Huang et al., 2010). Due to the lack of a significant plastic yielding phase in the quasi-brittle failure process, the load–displacement relationships were more suited to conducting the comparison among the fracture parameters. In this study, the predicted fracture load, fracture time, and apparent stiffness in the elastic phase were all compared to the experimental data to discuss the prediction accuracy between the two simulations. Furthermore, the comparisons of the fracture patterns between the simulations and experiments were also used to verify the conclusions obtained from the match of the load–displacement curves. The comparisons of the load–displacement curves and the fracture patterns stated that the simulation predicted by applying the minimum principal strain was in better agreement with the experimental results. The fracture parameters and the direction of the crack propagation in the simulation using the minimum principal strain were similar to the experimental data. Therefore, compared with the equivalent strain, the application of the minimum principal strain to control the element mechanical state was more suitable for simulating the cortical bone fracture under compression conditions.

The discrepancy in the predicted results must be caused by adopting different strain judging criteria because the structural and material parameters in the two simulations were identical. The cortical bone structures were subjected to an axial compression load. The minimum principal strain in the element should be the unidirectional strain similar to the loading direction, while the equivalent strain was calculated by considering the transverse, longitudinal, and shear strains (Onaka, 2010; Hambli and Allaoui, 2013). Thus, the intrinsic reason for the differences in the results may depend on whether the shear strain was considered and which strain was dominant during the compression process.

**FIGURE 6:**
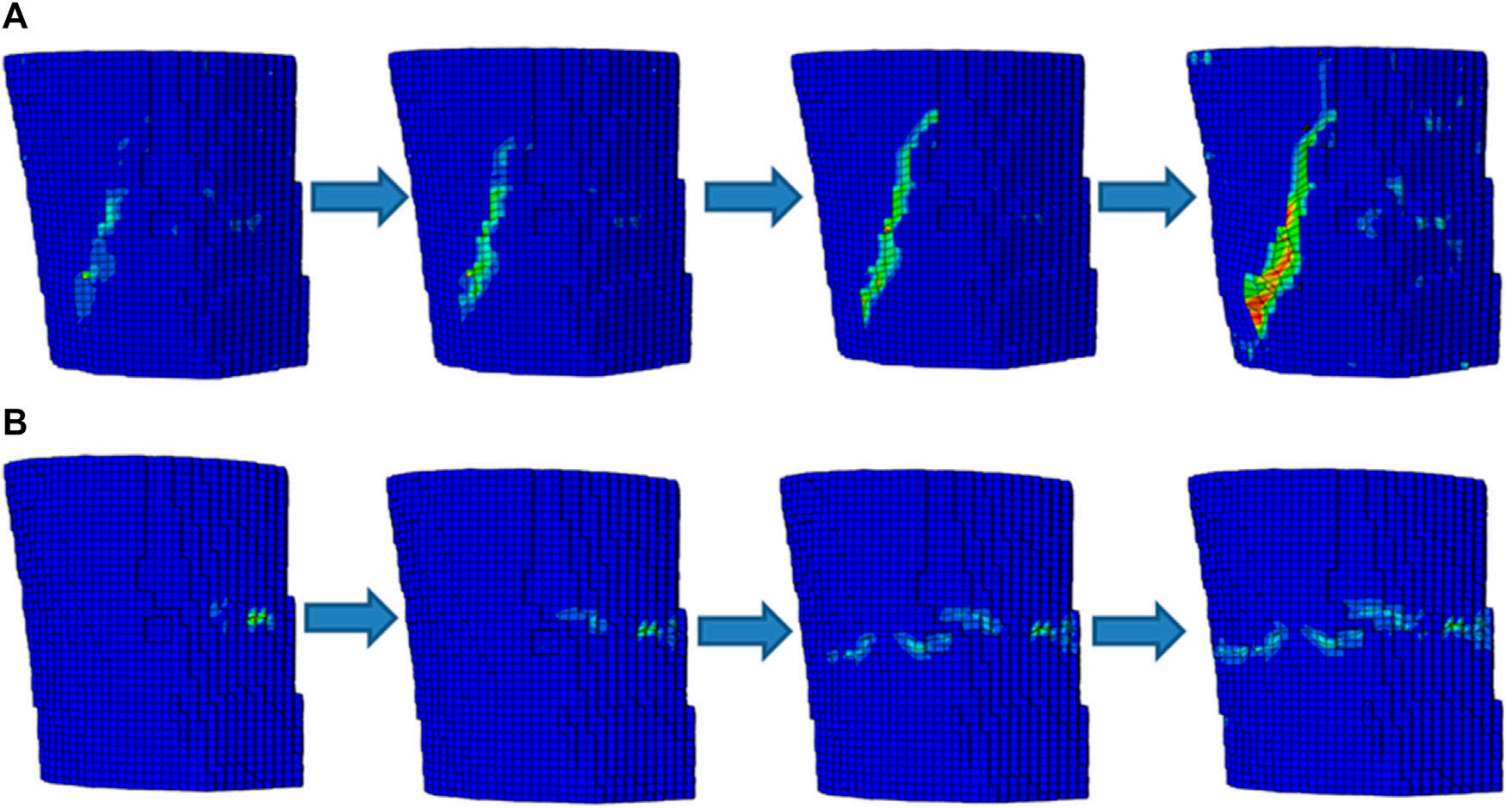
The schematic diagrams of the failure processes in the two simulations **(A)** Fracture simulation predicted by applying the minimum principal strain; **(B)** Fracture simulation predicted by applying the equivalent strain.

When element damage occurred, the differences in the crack initiation location appeared in the two simulations, as shown in Figure 6. The oblique crack, which was expressed at a certain angle to the loading direction, appeared in the simulation predicted by applying the minimum principal strain. This phenomenon was mainly because the minimum principal strain was unidirectional in the load direction, and the load direction may transmit at a certain angle to the osteon in the cortical bone specimen due to friction during compression (Hambli and Allaoui, 2013). However, the transverse crack, which was perpendicular to the loading direction, appeared in the simulation predicted by applying the equivalent strain, indicating that the shear strain in the element increased faster and dominated at the crack initiation stage (Feerick et al., 2013). Both the cracks then propagated with increasing compression. Once the crack initiation location is confirmed, the driving force will expand in the direction of the crack and will not easily change direction due to the limitation of the boundary condition (Zimmermann et al., 2009). Thus, one propagated along a certain angle to the loading direction, and the other propagated perpendicular to the loading direction. Because transverse propagation required crossing many osteons, which needed more energy than longitudinal propagation, the predicted results expressed a greater fracture load in the simulation using the equivalent strain than that of using the minimum principal strain.

It can be inferred that the equivalent strain was not suitable for judging the element mechanical status and performing the compressive fracture simulation for the cortical bone structure because the shear strain in the element increased faster than the longitudinal strain at the beginning of the loading and dominated the driving force at the crack initiation stage. Based on these, the innovation of this paper was to further improve the simulation accuracy of the cortical bone fracture under compression. The principal strain or equivalent strain was always selected as the criterion to judge the crack initiation and propagation in the element of the FE model (Dall’Ara and Tozzi, 2022; Hambli and Allaoui, 2013). However, various types of strain rise differently, even under the same loading condition (Dall’Ara et al., 2017). The inappropriate application of the strain judging criterion for performing fracture simulation may result in differences in the damage initiation and propagation processes, thus affecting the whole failure process. Therefore, the strain judging criterion suitable for simulating compressive fracture of the cortical bone structure was explored in this study, which may further improve the prediction accuracy of fracture simulation on the basis of previous simulations.

The fracture simulations in this study had several limitations. First, only compressive load was discussed, and the conclusions obtained in this study were only applicable to this loading condition. The cortical bone may also be subjected to other loads, e.g., bending and torsion in daily activity, and the change in the element strain in the cortical bone FE model may differ in various loading conditions. Therefore, the three-point bending simulation and experiment on the rat femur are planned to be conducted in the future to explore the suitable strain judging criterion in fracture simulation under bending load. Second, only the minimum principal strain in the element was considered because the cortical bone was subjected to a compressive load. Although the cortical bone structure was relatively simple and the bone unit aligned in the same direction as the loading direction, generating tensile strain in the element was also possible (Li et al., 2013; Dai et al., 2022). However, the accuracy of the simulation results in this study can be illustrated by comparing them with the experimental results. These indicated that the tensile strain generated in the cortical bone FE model under compressive load was relatively low, and the failure to consider the tensile strain did not have a substantial effect on the simulation results.

This study evaluated the prediction accuracy of the fracture simulations predicted by applying the minimum principal and equivalent strains. Experimental verification showed that the application of the minimum principal strain to control the damage and failure state in the element of the FE model could accurately simulate the failure process in the cortical bone structure under compression. Meanwhile, based on the comparisons of the load–displacement curves and fracture patterns, the reasons for the differences between the simulation using the equivalent strain and the experimental results were also revealed. Thus, this study provides a foundation for exploring the suitable strain judging criterion for cortical bone fracture under compression, which has the potential to improve prediction accuracy in the bone biomechanics field.

## Data Availability

The original contributions presented in the study are included in the article/supplementary material, further inquiries can be directed to the corresponding author.
